# Complete Genome Sequence of Streptococcus mutans Strain LAB761, Which Harbors Several Bacteriocin Loci, Isolated from a Caries-Active Child in Canada

**DOI:** 10.1128/MRA.01483-18

**Published:** 2019-01-10

**Authors:** Abdelahhad Barbour, Richard Yi He, Siew-Ging Gong, Céline M. Lévesque

**Affiliations:** a Faculty of Dentistry, University of Toronto, Toronto, Ontario, Canada; Louisiana State University

## Abstract

Streptococcus mutans LAB761 has been isolated from dental plaque collected from a child with severe caries. We report here the complete genome sequence of S. mutans strain LAB761, which has a chromosome of 2.0 Mb. The genome sequence reported herein contains several loci encoding double-glycine-motif peptides and lantibiotic and nonlantibiotic bacteriocins.

## Abstract (precis)

Streptococcus mutans LAB761 has been isolated from dental plaque collected from a child with severe caries. We report here the complete genome sequence of S. mutans strain LAB761, which has a chromosome of 2.0 Mb.

## ANNOUNCEMENT

Streptococcus mutans is an important human pathogen. It resides in the oral biofilm (dental plaque), and is one of the main causative agents of dental caries, the most prevalent streptococcal disease (1). The transmission of S. mutans usually occurs from mother to child via salivary transfer. However, detection of genotypes that are not shared with any household members suggest both horizontal and vertical routes of transmission (2). S. mutans possesses several virulence factors that contribute to its pathogenicity. These factors include its ability to adhere to the tooth surface as part of the multispecies biofilm community, to both produce and withstand highly acidic conditions, and to compete within the biofilm through the production of antimicrobial peptides called bacteriocins or mutacins (3, 4). S. mutans strain LAB761 was isolated from the supragingival plaque collected from facial and lingual smooth surfaces of primary maxillary incisors of a severe early childhood caries child age 3 years 4 months (REB protocol reference 32740). The plaque sample was plated on Mitis-Salivarius-Bacitracin agar supplemented with 20% sucrose using a spiral plater. Isolate LAB761 was verified as S. mutans by PCR (5) and 16S rRNA sequencing (6). Here, we present the complete genome sequence of this strain.

Strain LAB761 was cultivated in a 50-ml volume of Todd-Hewitt broth supplemented with 0.3% yeast extract at 37°C in air with 5% CO_2_ for 18 h without agitation. Genomic DNA was extracted using an in-house protocol. Briefly, cells were lysed with lysozyme (50 mg/ml at 37°C for 1 h), and proteins were digested with proteinase K (20 mg/ml at 37°C for 15 min) and precipitated with cold potassium acetate. The DNA was fished out using a sterile glass pipette and treated with RNase. DNA was quantified using the Quant-iT PicoGreen double-stranded DNA (dsDNA) assay kit (Thermo Fisher). Whole-genome sequencing was performed using the PacBio sequencing technology. The DNA library was prepared following the Pacific Biosciences 20-kb template preparation using the BluePippin size-selection system protocol. Qualified genomic DNA was fragmented using the Covaris g-TUBE device and then end-repaired to prepare SMRTbell DNA template libraries (with a fragment size of 15 kb to 50 kb) selected using a BluePippin system. Sequencing was performed using a Pacific Biosciences RSII sequencer using the MagBead one cell per well (OCPW) protocol at the Génome Québec Innovation Centre and Canadian Centre for Computational Genomics (McGill University, Québec, Canada). PacBio sequencing using 1 single-molecule real-time (SMRT) cell generated a total of 42,960 raw subreads of average length 12,149 bp, with an *N*_50_ value of 26,099 bp. Contig assembly was done using the HGAP workflow using default settings (7). The assembled genome had 246× genome coverage. The genome with a total size of 2,076,490 bp is composed of a single contig with a G+C content of 37.8%. Circularization of the contig at the overlapping ends was demonstrated using the Gepard software version 1.4 (8). Gene prediction and annotation were performed using RAST (9) and BLASTp (10). The genome annotation consisted of 1,841 protein-coding genes (CDSs), 64 tRNAs, and 5 rRNAs. The genomic information was analyzed to predict putative bacteriocin gene clusters and biosynthesis genes using the BAGEL4 (11) and antiSMASH 3.0 (12) Web servers with default search options. These pipelines predicted several genes for bacteriocin production and genes encoding uncharacterized double-glycine motif-containing peptides, in particular, genes related to mutacin Smb, mutacin IV, mutacin V, and mutacin VI. We also identified a locus of nine genes related to the production of the lantibiotic bacteriocin B-Ny266. Sequencing of multiple complete bacterial genomes is critical for evolutionary genomics. A comparative genomics approach will most likely be useful for a better characterization of the bacteriocin repertoire in S. mutans.

### Data availability.

The complete genome sequence has been deposited in GenBank under the accession number CP033199. Raw sequencing reads were deposited in the NCBI Sequence Read Archive (SRA) under accession number SRR8245044 and BioProject number PRJNA497888.
